# Ultrasound of Lipomatosis of Nerve Associated with Macrodactyly: ‘Spaghetti and Chocolate Cookie’ Appearance

**DOI:** 10.5334/jbsr.2849

**Published:** 2022-07-22

**Authors:** Kristin Francken, Tjeerd Jager, Johan Vanlauwe, Johan de Mey, Maryam Shahabpour, Michel De Maeseneer

**Affiliations:** 1 Department of Radiology, UZ Brussel, Brussels, BE; 2 Present address: Department of Radiology, Rijkevorsel, BE; 3 Department of Radiology, Aalsters Stedelijk Ziekenhuis, Aalst, BE; 4 Department of Orthopedic Surgery, UZ Brussel, Brussels, BE; 5 Free University Brussels, Brussels, BE

**Keywords:** Lipomatosis of nerve Fibrolipomatous hamartoma, US Macrodystrophia lipomatosa Macrodactyly, US

## Abstract

We studied the US imaging findings of lipomatosis of nerve and macrodystrophia lipoma in three patients.

Three patients were seen at three affiliated institutions with an enlarged digit. They all underwent US, subsequently followed by an MRI study.

The nerves showed marked enlargement and extension over a length of 9–16 cm. Digital branches were always involved. The appearance on cross section was an enlarged hyperechoic endoneurium with inlying thickened and hypoechoic fascicles. On transverse images this resulted in a ‘chocolate cookie’ aspect and in the long axis a ‘spaghetti-like’ image.

The US appearance of lipomatosis of nerve, not unlike MRI, is rather typical. With US, care should be taken in areas that are more difficult to assess as the sole of the foot, or where the ‘chocolate cookie’ appearance is not so obvious, such as the digits.

## Introduction

Macrodystrophia lipomatosa is a rare condition of unknown origin. It manifests as benign overgrowth of fibroadipose tissue, and bone in a digit or digits. This is most often associated with a peculiar nerve tumor, lipomatosis of nerve also previously termed fibrolipomatous hamartoma [1,2,3,4].

The pattern of changes, if one is not aware of the condition may be puzzling. In addition, the condition has been typically described in the median nerve territory and on MRI [1,2,3,4].

Lipomatosis of nerve is discussed by Enzinger and Weiss as a tumorlike lipomatous process involving peripheral nerves and their branches. Many previous case series have described the imaging of this condition [1,2,3,4]. To our knowledge, only a limited number of reports discussed ultrasound findings [5,6,7].

Diagnosis of the various tissue proliferations is mostly made on magnetic resonance imaging (MRI). US can, however, also evaluate these changes and especially the nerve enlargement, which is as we believe also typical on ultrasound.

We describe the ultrasound findings in three patients we encountered in clinical practice with lipomatosis of nerve causing macrodactily.

## Materials and Methods

In three affiliated institutions we encountered three patients with this condition. We present these three cases of MDL associated with fibrolipomatous hamartoma, in two cases involving atypical peripheral nerves. To the best of our knowledge, we did not find a report of ulnar nerve involvement. Ultrasound was performed in the patients. Findings were then subsequently correlated with MRI.

US imaging studies of three patients (two men and one woman) were obtained from three institutions. State of the art US systems were used using a 14 MHz or higher probe (Aloka F75; Canon Aplio 800). The patients were then subsequently imaged with MRI. Patient one and two were imaged using a Siemens 3-T (Siemens, Erlangen, Germany) and for patient three a GE 3-T (GE, Milwaukee, Wisconsin) was used. We analyzed clinical presentation and findings and morphological features on US and MRI including: size, shape and margins of the lesion as well as relationship to neighboring structures, the amount of fatty proliferation in the nerve, surrounding muscles, bones, and subcutaneous tissues.

## Results

The data from our study are presented in Tables 1 and 2. The patients were 5, 15, and 30 years old. The sites of nerve involvement were the ulnar and median nerve, and the medial plantar nerve in the foot.

**Table 1:** Clinical data in three patients.


PATIENT	AGE	SEX	AFFECTED NERVE	CLINICAL SYMPTOMS

1	5	F	Median nerve and digital branches for digit 3	Swelling of the palm of the hand and third finger, absence of pain

2	30	M	Medial plantar nerve and digital branches for digit 1	Macrodactyly first toe, unable to wear shoes

3	15	M	Ulnar nerve and digital branches for digit 5	Macrodactyly 5th finger, no pain


**Table 2:** Imaging findings in three patients on US.


PATIENT	SIZE	EXTENSION	OTHER FINDINGS	OTHER EXAMS

1	Length 9 cm; transverse diameter 1.5 × 0.5 cm	From proximal of the carpal tunnel into the digital nerves (digit 1,2 and 3)	Thickening subcutaneous fat and skin	US: ‘Spaghetti-like’ and ‘Coaxial cable-like’ appearance

2	Length 16 cm; transverse diameter 1.8 × 1.6 cm	From proximal medial plantar nerve into the digital nerves (digit 1 and 2)	Thickening subcutaneous fat and skin hypertrophic bone structures (metatarsal and phalangeal bones)	US: excessive subcutaneous fat. Thickened nerve showed coaxial cable and spaghetti aspect

3	Length 16 cm; transverse diameter 1.5 × 0.9 cm	From distal forearm into the digital nerves (digit 4 and 5)	Enlargement of subcutaneous fatty tissue	US: ‘Spaghetti-like’ and ‘Coaxial cable-like’ appearance


**Figure 1:**
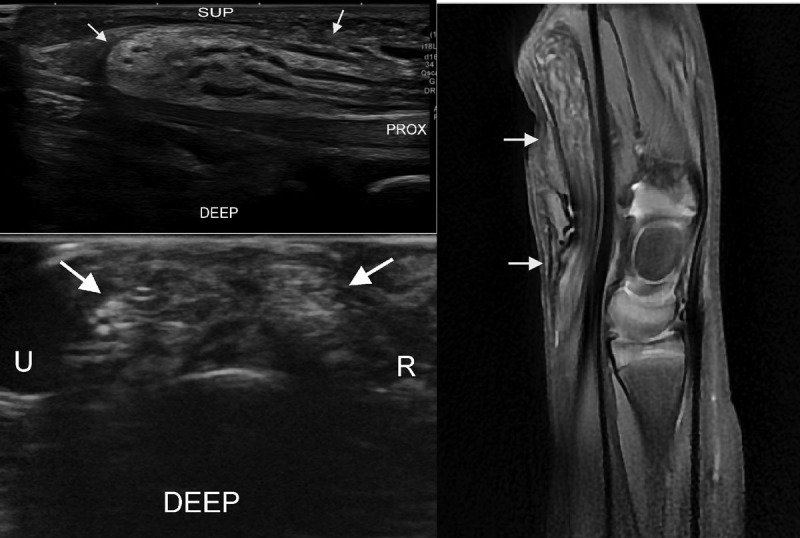
Left top image. Long axis US image of patient 1. The extensive involvement of the median nerve is seen beyond the carpal tunnel. The spaghetti like appearance can be appreciated (arrows). Endoneurial fat is hyperechoic, the nerve fascicles are hypoechoic and thickened. Left bottom image. Transverse US image of third digit. Note marked increase of fatty tissue in location of digital nerve. The coaxial cable aspect is less obvious here. Right image. Sagittal PD-w MR with FS. Note the extensive ‘spaghetti-like image’ (arrows) comparable to US.

**Figure 2:**
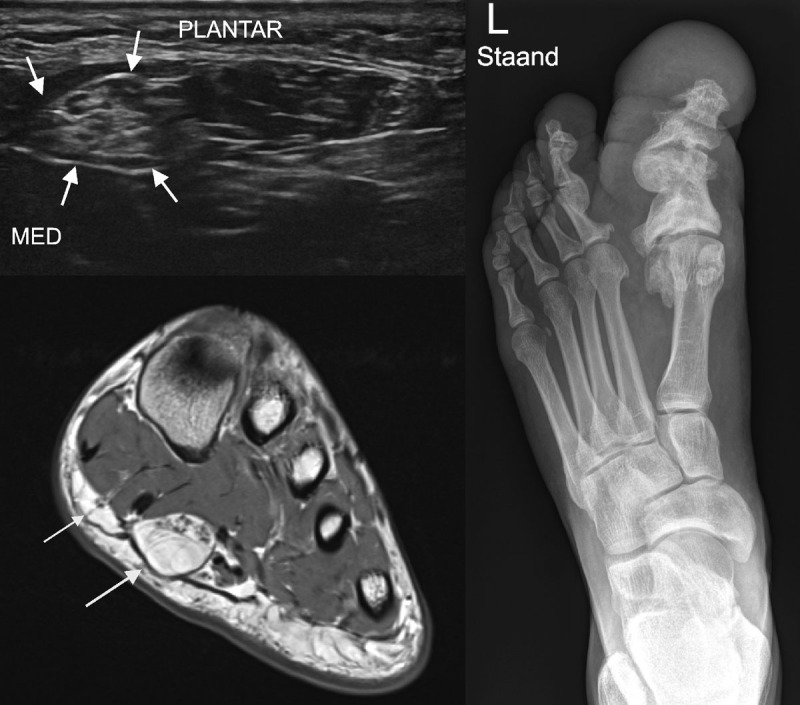
Left top image. US. Enlarged medial plantar nerve (arrows) in short axis next to flexor digitorum brevis in patient 2. Left bottom image. MR image in exactly the same location. The thickened medial plantar nerve can be seen as well as the medial hallucal nerve (arrow). Right image. Radiography. Macrodactyly in patient 2. The hallux is markedly enlarged with an increase in soft tissue and bony overgrowth in an irregular fashion.

US demonstrated the increased subcutaneous fat as well as hypoechoic streaks indicating an increase in fibrous thickened septations in the soft tissues. Lipomatosis of nerve (fibrolipomatous hamartoma) was shown as a generally rounded structure (Figures 1, 2, 3). In the short axis fatty proliferation was hyperechoic and the nerve fascicles had a hypoechoic appearance. The fascicles were thickened, somewhat irregularly, compared to normal nerve fascicles. Of note, some difficulty was encountered in the sole of the foot due to the more limited penetration by the foot skin, and the poor differentiation from surrounding fat that is prominent in the sole of the foot (Figure 2). The delineation of the nerve from adjacent tissues was also rather poor, and no true capsule was seen. This made the diagnosis quite difficult in for example the digital branches (Figures 1 and 3). We prefer to describe the transverse appearance rather as ‘a chocolate cookie’ aspect given the presence of irregular shape of the fascicles. This appearance was less obvious in the digital branches, rather an increase of hyperechoic fatty tissues in the expected location of the digital nerves was seen (Figures 1 and 3). Also, beyond the carpal tunnel, and the MTP joint, the nerves became more oval, somewhat flattened, and the coaxial cable like appearance was less obvious. The nerves tended to show a course in multiple directions. In the long axis, the nerve was also seen to present a marked thickening. The endoneurial fat was also hyperechoic, and the fascicles were hypoechoic. For this appearance the MRI derived description of ‘spaghetti-like’ image was suitable (Figures 1 and 2). Nerve enlargement over a distance of 9 – 16 cm was seen. All the three patients demonstrated extension of the lesions into the digital nerves.

**Figure 3:**
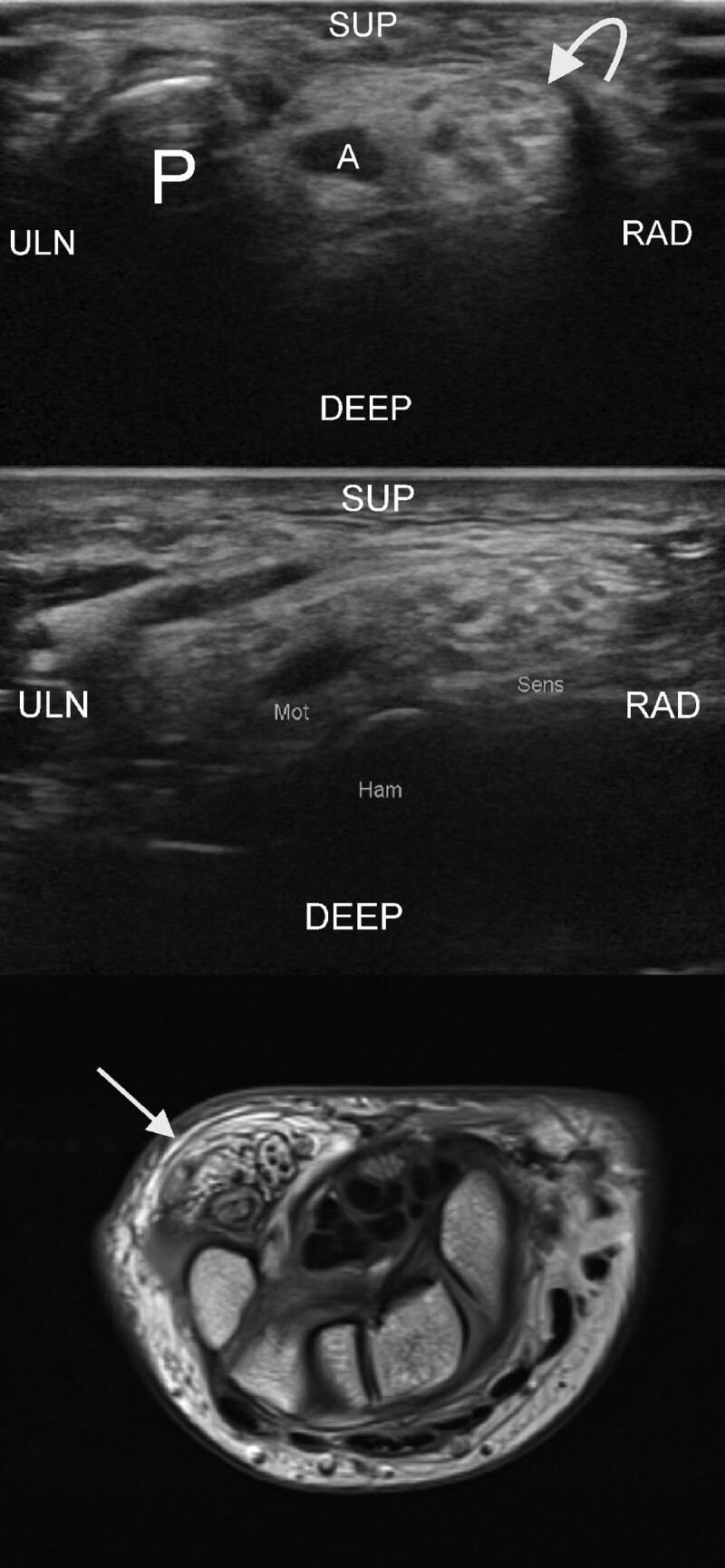
**A.** Patient 3 Top image. Transverse ultrasound image of the canal of Guyon in patient 3. Note pisiform bone (P), adjacent to it is the ulnar artery, and next to it the enlarged ulnar nerve. Middle image. Transverse ultrasound image at the level of the hook of the hamate. Note that the motor branch in the pisohamate hiatus appears relatively normal (it has been suggested motor nerves are less affected). The sensory branches are a bit flattened but the coaxial cable aspect is still obvious. Bottom image. MR imaging. Ulnar nerve in the canal of Guyon. Note markedly thickened nerve. Coaxial cable aspect is less obvious and appearance is more swirly-looking. (arrow)

**Figure 3:**
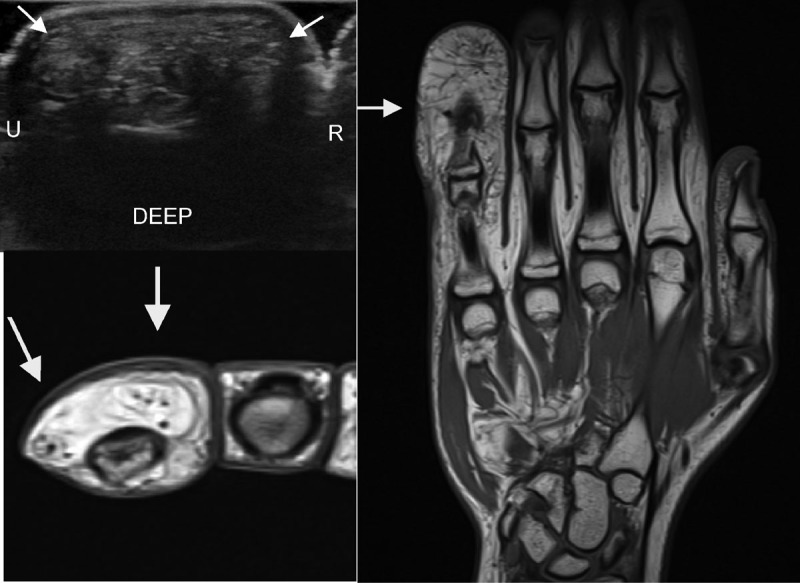
**B.** Patient 3. Left top and bottom image. US and MRI. Marked enlargement of the digital nerves. Note there is predominantly fatty proliferation and the coaxial cable aspect is not obvious (arrows). Right image. MRI. Marked enlargement (macrodactyly) of the little finger is seen.

MRI confirmed the excess fibrofatty and bone tissue (Figure 1). The fibrous strands within the fatty tissue were seen as low-signal-intensity linear streaks on PD-w images. The thickened nerves were also seen, exhibiting characteristics as described in the literature [1,2,3,4,5,6,7].

## Discussion

Lipomatosis of nerve (or fibrolipomatous hamartoma) and macrodystrophia lipomatosa is an uncommon tumorlike process of peripheral nerves. It is characterized by an increase in all mesenchymal elements, particularly fibroadipose tissue, but also bone. The area of predilection is the median nerve [1,2,3,4]. There is no gender predilection.

The disorder is congenital in origin, but not hereditary [1,2,3,4]. The exact etiology of the disorder is unknown. There are three probable mechanisms: abnormal nerve proliferation, abnormal blood supply or trauma [8].

Lipomatosis of nerve can be associated with bone overgrowth, in about one third of cases it is associated with macrodactyly. This is termed as macrodystrophia lipomatosa [1,2,3,4].

In this paper, we focused on the US diagnosis of lipomatosis of nerve [5,6,7]. The appearance of fibrofatty tissue in the digits in macrodystrophia lipomatosa has been described previously on radiography and MRI.

In our study the patients presented with an enlarged digit, without pain, but obviously with a disfigured cosmetic aspect and problems for wearing shoes in patient 2.

In our series the tumors were quite large both in short and long axis with a length of 9 cm in one patient and 16 cm in the two other patients.

**Figure 4:**
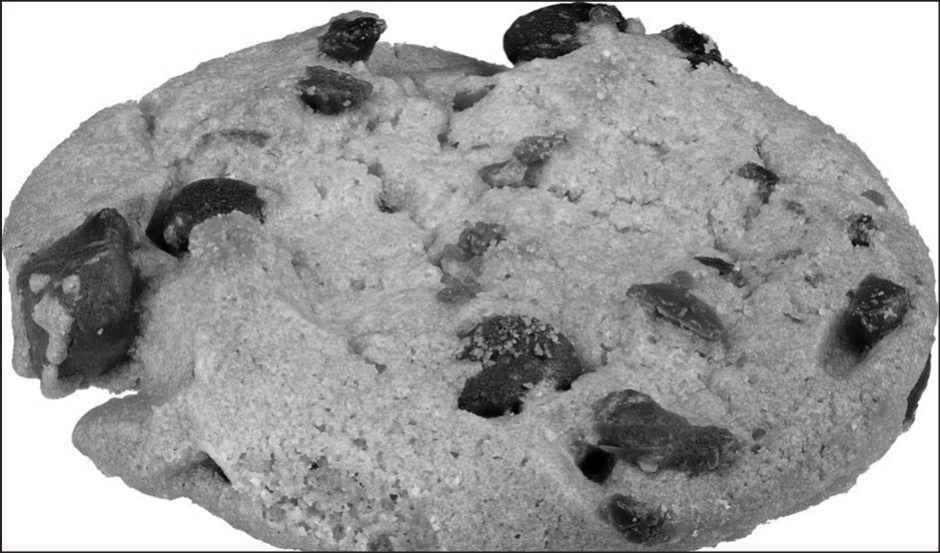
Photograph of a ‘chocolate cookie’ resembling the transverse imaging on US of lipomatosis of nerve.

On US the increase of fibrofatty tissue occurred predominantly in the enlarged digits. Increased septations were seen in the subcutaneous fat. The nerves were seen to be markedly thickened. Endoneurial fat was increased with a hyperechoic aspect, whereas the nerve fascicles were also enlarged but with a hypoechoic aspect. The fascicles are not unlike those seen in a normal nerve, except thickened, but the increase in endoneurial fat is normally not observed. Of note the nerves are not markedly encapsulated. Not unlike the findings on MRI, the short axis view could be compared to a coaxial cable, although we prefer the ‘chocolate cookie’ term, and the long axis view to a ‘spaghetti like image’ (Figure 4). In the digits this appearance was less obvious and an increase in fatty tissue was seen in the expected location of the nerve. The thickened fascicles were less obvious in this location. Also, in the sole of the foot the thickened nerve could potentially be overlooked by the poorer penetration of US in the foot.

Overall, our findings show similarity with described MRI findings of lipomatosis of nerve [3].

Other conditions can cause macrodactyly and enlarged limbs or nerves. Lipomatosis of nerve is however not seen in these diseases. US features of these conditions have also only been sparsely described, most descriptions involving MRI. Neurofibromatosis type 1 with plexiform neurofibromas is probably the most common condition coming to mind. The aspect is quite different. The neurofibromas have a clustered hypoechoic cystic aspect along the course of the nerves. In Klippel Trenaunay vascular overgrowth causes the enlarged limb but as mentioned the overgrowth is limited to vascular structures, which can be documented with doppler US.

Our study has some limitations. First, the investigation was conducted at three institutions, and a systematic approach was absent, each MSK radiologist using his own examination technique. However, all radiologists were experienced in MSK US. Further, our series was limited. However, this is an uncommon pathology. Nevertheless, the US features were consistent, although location and extend was variable. Also, the US and MR systems were different, but all were state of the art. We also did not use panoramic US imaging which could have been useful. We did not have any histological analysis, but surgery with these lesions is not so typical as the nerves would have to be sacrificed, resulting in neurologic deficits [9,10].

The diagnosis of macrodystrophia lipomatosa is generally not difficult although some conditions exist also causing an enlarged limb. If lipomatosis of nerve is associated on US a ‘chocolate cookie’ or ‘coaxial cable’ aspect can be seen in the short axis and a ‘spaghetti like’ appearance in the long axis, findings not unlike MRI. Care has to be taken to identify the process in digital branches and in difficult locations, such as the sole of the foot.

In conclusion, we describe the US appearance which one should be aware of to make a correct diagnosis. In the transverse plane the endoneurium is hyperechoic and there are inlying hypoechoic fascicles. In the long axis a spaghetti like image is seen.
